# Time and frequency analysis of daily-based nexus between global CO_2_ emissions and electricity generation nexus by novel WLMC approach

**DOI:** 10.1038/s41598-024-54245-z

**Published:** 2024-02-14

**Authors:** Mustafa Tevfik Kartal, Talat Ulussever, Ugur Korkut Pata, Serpil Kılıç Depren

**Affiliations:** 1 Department of Banking and Finance, European University of Lefke, Lefke, Northern Cyprus Türkiye; 2 Adnan Kassar School of Business, Lebanese American University, Beirut, Lebanon; 3 Clinic of Economics, Azerbaijan State University of Economics (UNEC), Baku, Azerbaijan; 4 Economics and Finance Department, Gulf University for Science and Technology, Mubarak Al-Abdullah, Kuwait; 5 Center for Sustainable Energy and Economic Development (SEED), Research Fellow, Gulf University for Science and Technology, Mubarak Al-Abdullah, Kuwait; 6 Department of Economics, Osmaniye Korkut Ata University, 80000 Merkez, Osmaniye Türkiye; 7 Department of Statistics, Yildiz Technical University, Istanbul, Türkiye

**Keywords:** CO_2_ emissions, Electricity generation, High-frequency data, Globe, Disaggregated analysis, WLMC, Climate-change mitigation, Energy policy, Energy supply and demand

## Abstract

The studies have focused on changes in CO_2_ emissions over different periods, including the COVID-19 pandemic. Even if CO_2_ emissions are temporarily reduced during the pandemic according to annual figures, this may be misleading. Considering annual figures is important to understand the overall trend, but using data with much higher frequency (e.g., daily) is much better suited to investigate dynamic relationships and external effects. Therefore, this study comprehensively analyzes the association between CO_2_ emissions and disaggregated electricity generation (EG) sources across the globe by employing the novel wavelet local multiple correlation (WLMC) approach on daily data from 1st January 2020 to 31st March 2023. The results demonstrate that (1) based on the main statistics, daily CO_2_ emissions range between 69 MtCO_2_ and 116 MtCO_2_, indicating that there is an oscillation, but no sharp changes over the analyzed period. (2) based on the baseline regression using the dynamic ordinary least squares (DOLS) approach, the constructed estimation models have a high predictive ability of CO_2_ emissions, reaching ~ 94%; (3) in the further analysis employing the WLMC approach, there are significant externalities between EG resources, which affect CO_2_ emissions. The results present novel insights about time- and frequency-varying effects as well as a disaggregated analysis of the effect of EG on CO_2_ emissions, demonstrating the significance of the energy transition towards clean sources around the world.

## Introduction

Anthropogenic activities have caused various negative environmental effects (e.g., global warming and climate change), mainly due to high energy consumption, especially fossil-based ones. Given these effects, all parties have paid attention to the environmental issues^1–3^. Scholars have searched for the causes of environmental degradation by considering various factors, time intervals, and econometric approaches to propose policy measures either as a possible solution or as a contribution to curbing environmental degradation^4^. Looking at the contemporary literature, it can be concluded that there is an increasing trend in greenhouse gas emissions, with CO_2_ emissions being the largest contributor in this context^5,6^. Therefore, one of the main concerns of all economic actors is to deal with CO_2_ emissions so that they are either not emitted or decreased.

Most research in the literature has focused on CO_2_ emissions using low-frequency (e.g., annual) data. However, the use of low-frequency data can be misleading because it only considers aggregate conditions over time and neglects the dynamic changes in the nexus over much longer periods and frequencies^7,8^. High-frequency data can address such issues and provide much more novel insights. Using data at an aggregate level can also prevent the understanding of externalities between various factors^9^. Since fossil fuels and clean energy sources are substitutes for each other, a change in either factor can have an externality on the other. In addition, changes in sub-components of fossil fuels and clean energy sources may cause further externalities on other sub-components either in other groups or in the same group. For these reasons, the use of higher-frequency and disaggregated level data may be much more appropriate when examining the dynamic nexus and externalities between CO_2_ emissions and EG at the global scale.

The lack of high-frequency (i.e., low-latency) data for global CO_2_ emissions directly related to activities has been an important issue for estimations until recently, as such a lack forces researchers to use some alternative factors as a proxy for CO_2_ emissions. However, it is now possible to work with near-real-time data on CO_2_ emissions^10,11^. The same condition applies to the EG, which is one of the main drivers of global CO_2_ emissions, as representing energy consumption^12^. In the current literature, a few studies have considered this near-real-time data as high-frequency (daily) data in predicting CO_2_ emissions at the country or global level^11,13,14^. There are some studies, that use daily data, but many of them have used relatively old data, which do not consider the recent issues (e.g., post-pandemic periods, higher geopolitical tensions, and the current energy crisis). From this point of view, then, there is a gap in the literature.

To fill this gap, this study investigates the role of the EG on CO_2_ emissions using the available up-to-date data from January 1, 2020, to March 31, 2023. The study focuses on five different models and applies the novel WLMC approach to uncover time- and frequency-varying associations. Such a comprehensive methodological basis enables researchers to examine the dynamic nexus and externalities between global CO_2_ emissions and the EG in depth. Taking into account the most recent daily data and thus the most up-to-date information, as well as socio-economic issues (e.g., the COVID-19 pandemic, energy crisis, and geopolitical issues), the study appraises the profound link between global CO_2_ emissions and the EG.

Following a comprehensive methodological approach, the study shows that there are no sharp daily changes in global CO_2_ emissions for the period examined, although there is an oscillation. The results present that the models have a high model performance criterion (up to ~ 94%) for predicting CO_2_ emissions considering various EG combinations. The WLMC analysis also demonstrates that there are significant externalities between the sub-components of EG resources that directly affect CO_2_ emissions and that the effect of EG course on CO_2_ emissions shows a time and frequency-varying trend. By examining the effect of EG on CO_2_ emissions at a global scale and considering times and frequencies with daily data, the study provides novel insights into the literature.

The second section of the study demonstrates the progress of global CO_2_ emissions and the EG. The third section of the study explains the methods. The fourth section of the study reports on the empirical results. The last section of the study discusses and concludes with policy measures.

## Progress of global CO_2_ emissions and EG

### Global CO_2_ emissions

**Figure 1:**
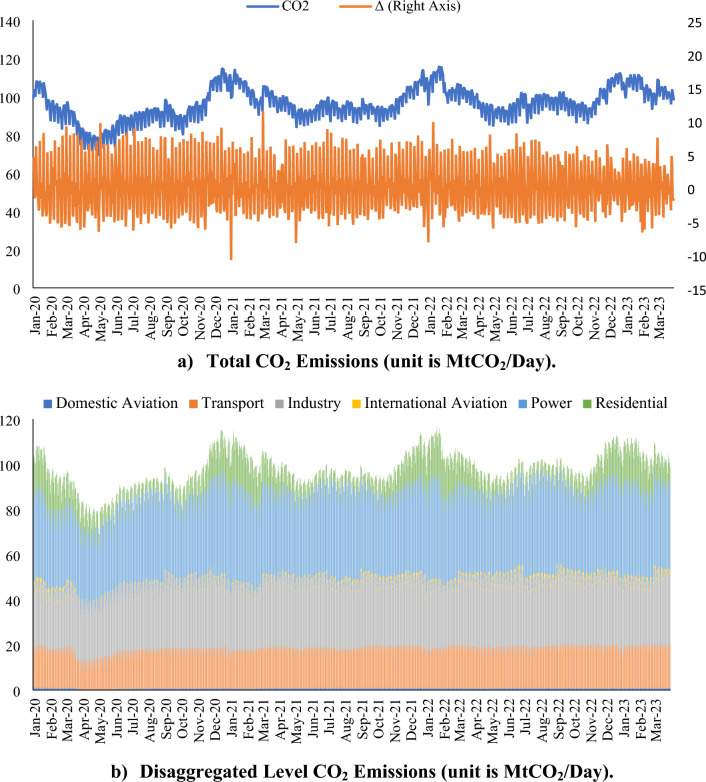
Progress trend of daily global CO_2_ emissions^15^.

Based on the statistics^5^, total CO_2_ emissions amounted to 34.1, 32.1, and 33.9 million tons in 2019, 2020, and 2021, respectively. This corresponds to a change of − 0.2%, − 5.9%, and + 5.6% compared to the previous year. Global CO_2_ emissions have shown an unpredictable decrease in global CO_2_ emissions in 2020, to which the pandemic has also contributed^11^. However, the downward trend has reversed with the easing of the pandemic measures. It is therefore important to consider diurnal changes for a much more comprehensive examination of CO_2_ emissions. Figure 1 demonstrates the trend in global CO_2_ emissions.

On average, 91.41 MtCO_2_ was emitted daily in 2020. This figure increased to 97.29 MtCO_2_ in 2021, 98.97 MtCO_2_ in 2022, and 104.09 MtCO_2_ in the first 3 months of 2023 (for more details on global CO_2_ emissions, see Supplementary Fig. 1; Supplementary Table 1). Although there is a temporary reduction in the first half of 2020 due to the COVID-19 pandemic measures at the level of total CO_2_ emissions (the low point is 69 MtCO_2_/day around 2022 May), this case reverses and total CO_2_ emissions continue to increase on average. As far as sectoral CO_2_ emissions are concerned, the same picture emerges again. In detail, the power sector causes the highest CO_2_ emissions, followed by industry, transport, residential, international1 aviation, and domestic aviation sectors, respectively. Although there are some fluctuations at both overall and sector levels, there is unfortunately no permanent reduction in global CO_2_ emissions.

### Global EG

**Figure 2:**
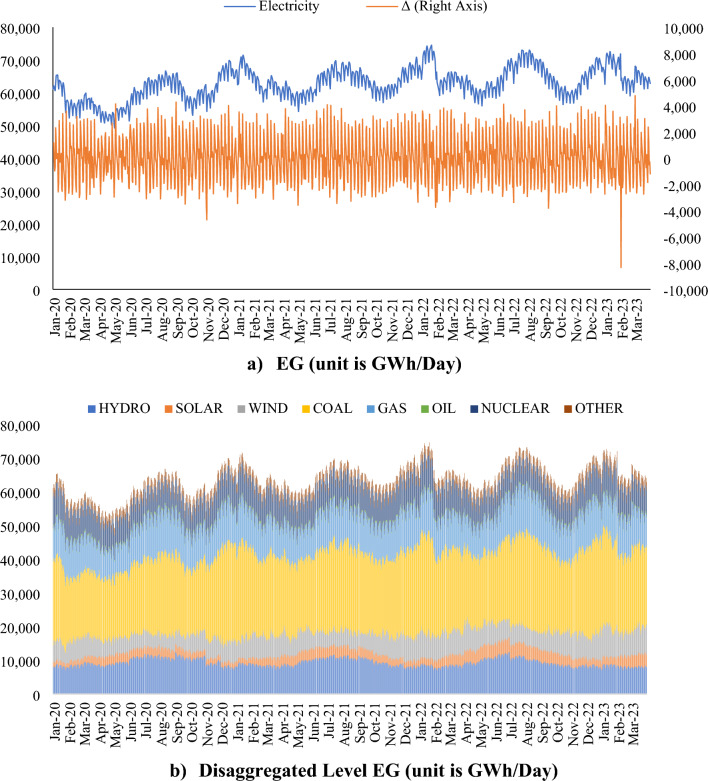
Progress trend of daily global EG^15^.

Global EG is considered to be one of the most important drivers of global CO_2_ emissions, and Fig. 2 shows the development of global EG.

On average, 59.5 TWh/day per day in 2020. This figure increased to 63.5 TWh/day in 2021, 65 TWh/day in 2022, and 65.9 TWh/day in the first 3 months of 2023 (for more details on global EG and its sub-components, see Supplementary Fig. 1; Supplementary Table 1). There is a temporary reduction in the first half of 2020 (the low point is 49 TWh/day around May 2022), which is due to the contribution of measures to combat the COVID-19 pandemic at the total EG level. However, this reduction is not permanent, but reverses, meaning that the total EG has continued to rise on average. The same picture emerges for source-based EG. Specifically, coal (37%) is the most important source of EG, followed by natural gas (17%), hydro (15%), nuclear (13%), wind (10%), solar (4%), and oil (1), in that order. Although there are some fluctuations on either the total or source-based level, no permanent decrease in EG can be observed. For the period between 2020/1 and 2023/3, fossil, renewable, nuclear, and other sources have a share of 55%, 29%, 13%, and 4% of total EG.

### Nexus between global CO_2_ Emissions and EG

Although there was a relative decline in both global CO_2_ emissions and global EG in the first half of 2020 due to the pandemic, this trend has reversed in later periods. As is known, various sources have been used as proxies for electricity generation, which ultimately affect global CO_2_ emissions. The correlation analysis presented in Supplementary Table 2 shows that although there are various sources, including clean ones, some of them have a negative correlation with global CO_2_ emissions. Only hydro (− 0.30) and solar (− 0.22) sources have a negative correlation, while all other sources have a positive correlation with CO_2_ emissions. A further correlation analysis, which is presented in Supplementary Table 3 for the sub-periods, illustrates that hydro, solar, and renewable energies show a negative correlation in 2020, 2021, and 2022. In the first three months of 2023, however, only solar energy shows a negative correlation. The correlation between CO_2_ emissions and EG sources varies depending on the sources and sub-periods. Therefore, it can be claimed that the nexus between global CO_2_ emissions and global EG has a varying structure. However, such an assertion requires a much more detailed analysis by applying further econometric methods.

## Methods

To investigate the nexus between global CO_2_ emissions and global EG, a multi-stage empirical methodology is applied, which is presented in Supplementary Fig. 2. First, the data for global CO_2_ emissions and EG are collected from the Carbonmonitor^15^. Descriptive statistics and correlations between the variables are then examined, and the characteristics of stationarity and linearity of the variables are investigated to select the appropriate econometric approaches. In the next stage, cointegration relationships are estimated and baseline regression results are examined with the DOLS approach. In the final stage, the WLMC analysis is carried out to empirically examine the nexus between daily CO_2_ emissions and EG at a global scale. Table 1 presents the information about the variables.

**Table 1:** Details of the variables.

Symbol	Definition	Unit
CO_2_	CO_2_ emissions*	MtCO_2_/day
FOSSIL	EG from fossil fuels (coal, natural gas, oil)	GWh/day
RENEW	EG from renewable energies (hydro, solar, wind)	
NUCLEAR	EG from nuclear	
HYDRO	EG from hydro	
SOLAR	EG from solar	
WIND	EG from wind	
COAL	EG from coal	
GAS	EG from natural gas	
OIL	EG from Oil	

*The dependent variable. All variables are related to the global level.

Taking into account the variables explained above, five different estimation models are utilized to examine the varying nexus between the variables, which are shown in Table 2.

**Table 2:** Details of the models.

Number	Name	Details of included variables
1	Main 1	CO_2_ = f (HYDRO, SOLAR, WIND, COAL, GAS, OIL)
2	Main 2	CO_2_ = f (FOSSIL, RENEW, NUCLEAR)
3	Fossil	CO_2_ = f (COAL, GAS, OIL)
4	Renewable	CO_2_ = f (HYDRO, SOLAR, WIND)
5	Mixed	CO_2_ = f (COAL, HYDRO, NUCLEAR)

Model 1 includes all sub-components of fossil and renewable energy sources so that the nexus between all sub-components and CO_2_ is predictable. This model therefore only refers to the information included in the baseline regression and is not considered in the further analysis. In Model 2, the most important main components of EG are considered. In Model 3, only fossil sources are used. In Model 4, only renewable sources are considered. In Model 5, the highest share of the sources in each group (i.e., fossil, renewable, and nuclear) is included.

Considering five different models and applying a novel WLMC approach with the daily high-frequency dataset, the study presents insights into the nexus between CO_2_ emissions and EG on a global scale.

## Results

### Baseline regression

Considering the data characteristics (see Supplementary Table 4) and the cointegration nexus (see Supplementary Table 5) between the variables for the constructed models, baseline regression models are first estimated using the DOLS approach, and the results are reported in Supplementary Table 6.

According to the baseline results, there are important nexus between global CO_2_ emissions and global EG. The effect of global EG on global CO_2_ emissions is statistically significant even with variations (i.e., different models). The constructed models have a high predictive power for CO_2_ emissions, reaching ~ 94% in the best model. Thus, it can be stated that global EG is a significant predictor of global CO_2_ emissions and can be used for further empirical investigations.

### WLMC approach

Having demonstrated the significant effect of global EG on global CO_2_ emissions through the baseline regression estimates, a further analysis is conducted to investigate the time and frequency-varying effect of global EG on global CO_2_ emissions. To this end, the WLMC approach is applied to the models constructed at disaggregated levels (i.e., Models 2–5), which are detailed in Table 2.

**Figure 3:**
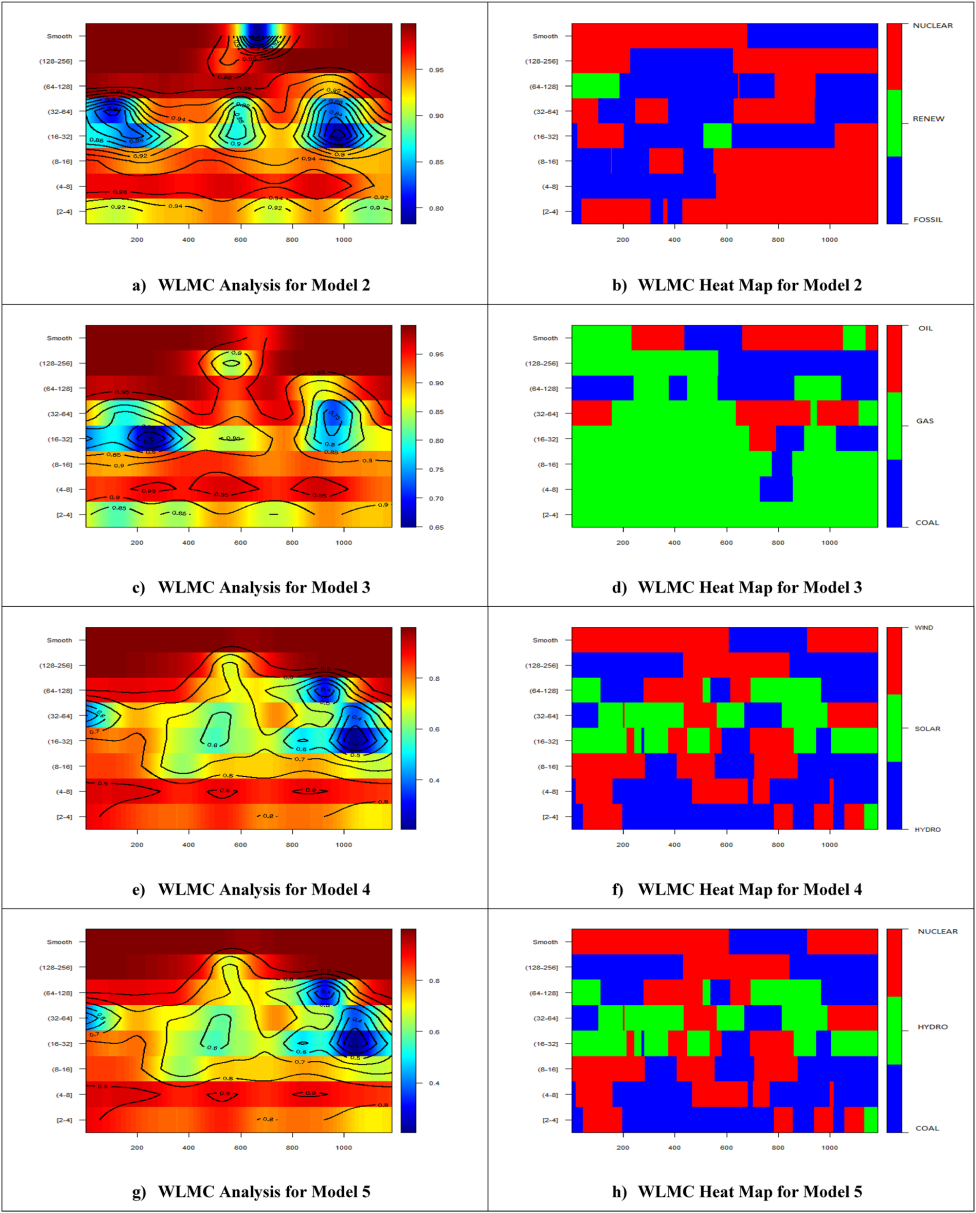
WLMC results for models.

In all WLMC graphs, the x-axis denotes the daily times from January 1, 2020, to March 31, 2023, and the y-axis denotes the frequency from low to high. Further explanations of the frequencies and the exact timelines of the observations can be found in Supplementary Tables 7 and 8, in that order. The plots in the left panel of the WLMC graphs present the four-variate cases of the EG sources considered in a specific model (i.e., Models 2–5) in terms of their effects on CO_2_ emissions across various times and frequencies. The plots in the right part of the WLMC graphs denote the heat map (i.e., the higher importance) of the EG sources considered in a specific model (i.e., Models 2–5) in terms of their effects on CO_2_ emissions across various times and frequencies. The results of the WLMC approach are summarized in Fig. 3.

Model 2 considers FOSSIL, RENEW, and NUCLEAR as EG sources, which are the main principal components of EG, to analyze the effect of global EG on global CO_2_ emissions. According to Fig. 3a, there is a positive nexus between global CO_2_ emissions and EG from different sources, which implies that an increase in global EG from FOSSIL, RENEW, and NUCLEAR causes an increase in global CO_2_ emissions. The increasing effect is much higher at lower and higher frequencies, reaching at most 0.98, while the effect is relatively lower at middle frequencies around 0.8, especially around the first quarter of 2020 and the third quarter of 2022. Looking at the externalities among FOSSIL, RENEW, and NUCLEAR in Fig. 3b, it can be seen that nuclear is the leading EG at lower frequencies and fossil EG is leading at higher frequencies. Overall, nuclear EG is more important until Q3 2021, but thereafter, fossil EG has a leading role in terms of the effect of EG on CO_2_ emissions at a global level.

Model 3 considers COAL, GAS, and OIL as EG sources to investigate the effect of global EG on global CO_2_ emissions. Figure 3c shows that there is a stronger positive correlation between global CO_2_ emissions and EG from various fossil sources, which implies that an increase in global EG from COAL, GAS, and OIL causes an increase in global CO_2_ emissions. The stimulating effect is much higher at lower and higher frequencies, reaching 0.95; however, it is relatively lower at middle frequencies around 0.7, especially in the first half of 2020 and the third quarter of 2022. Figure 3d demonstrated the externalities between COAL, GAS, and OIL. It can be seen that natural gas EG occupies a leading position across various frequencies. The importance of both oil EG and coal EG has recently increased at higher frequencies with the contribution of the energy crisis. Oil holds the leading position in the first three quarters of 2022 and the first months of 2023.

Model 4 considers HYDRO, SOLAR, and WIND as EG sources to investigate the effect of global EG on global CO_2_ emissions. As shown in Fig. 3e, renewable EG has a positive and strong impact on global CO_2_ emissions. It is much higher at lower and higher frequencies, reaching 0.9, but the effect is significantly lower at middle frequencies around 0.3, especially around the third quarter of 2022. From the externalities perspective in Fig. 3f, it can be seen that hydro EG has a leading position at low frequencies followed by solar EG at middle frequencies and wind EG at higher frequencies. Overall, wind EG is the dominant renewable source, with the exception of the period between 2021/8 and 2022/5, where hydro EG takes the lead position.

Model 5 considers COAL, HYDRO, and NUCLEAR as EG sources. It is a mixed model constructed based on the highest share of EG sources in each group (i.e., fossil, renewable, and nuclear), to investigate the effect of global EG on global CO_2_ emissions. As shown in Fig. 3g, there is a positive nexus between global CO_2_ emissions and EG from these sources. The increasing effect is much higher at lower and higher frequencies by 0.9, while the effect is relatively lower at middle frequencies around 0.3, especially around the first quarter of 2020 and the third quarter of 2022. From the externalities perspective in Fig. 3h, it can be seen that coal EG leads at lower frequencies, hydro EG is superior at middle frequencies, and nuclear EG is generally stronger across frequencies at various times. Overall, nuclear EG is the dominant source except for the period between 2021/8 and 2022/5 where coal EG is the leading source.

The WLMC approach results demonstrate that, in general, there is an increasing effect of the disaggregated level of global EG on global CO_2_ emissions. However, the effect differs depending on time, frequency, and constructed models.

## Discussion and implications

The results of the study reveal that there are some variations in global CO_2_ emissions between the days. However, it is not a permanent decrease that is consistent with studies in the literature^16–20^. Instead, this could be because of the various causes (e.g., seasonality, weather conditions, technological changes) that have affected anthropogenic activities (e.g., electricity consumption). The recent energy crisis has also changed the nexus between global CO_2_ emissions and global EG, which was analyzed using four different models.

The correlation analysis provides initial indications of the varying effects of global EG on global CO_2_ emissions. The baseline regression estimates reveal that global EG sources are highly related to global CO_2_ emissions, which have different impacts based on the coefficient estimates and R^2^ values of the constructed models. The WLMC approach demonstrates that there are significant externalities between EG resources even when they occur in the same sub-groups (either in fossil or renewable). For example, fossil EG is the leading source among fossil, renewable, and nuclear sources. Oil EG is the leading source among fossil sources, while wind EG is the most significant source among renewable sources. Furthermore, the nexus between global CO_2_ emissions and global EG is dynamic and exhibits a time- and frequency-varying structure. Therefore, the results have certainly proven the changing effect of global EG on global CO_2_ emissions depending on time, frequency, and constructed models as well as the sources considered in the models.

Importantly, although there are changing effects of global EG on global CO_2_ emissions at various times, it cannot be said that the current energy crisis has a certain (i.e., increasing or decreasing) effect. Rather, it has caused a continuous change in EG sources. For this reason, total global EG and CO_2_ emissions have remained almost the same or even increased on average in the period between 2020 and 2023, although there are some fluctuations between days.

The current energy crisis has affected the clean energy transition because countries have tried to replace cuts in natural gas supply for EG with other alternatives^4,21^. CO_2_ emissions from EG have not fallen permanently as countries have prioritized energy security and economic growth in the recent energy crisis. Therefore, fossil fuels still have a high share in the total EG and their share has even increased recently due to the effects of the energy crisis as they cause externalities among the possible sources. There is still a long way to ensure energy transition to clean energy sources to curb global CO_2_ emissions resulting from global EG, considering the results of the study based on the novel dataset and econometric methodology. Based on these results and the discussion, it is recommended that countries facing an energy crisis should make further efforts to support the transition to clean energy and prevent the increase of fossil fuel use in the EG.

### Supplementary Information

Supplementary Information.

## Data Availability

The datasets used and/or analyzed during the current study is available from the corresponding author (Mustafa Tevfik Kartal) on reasonable request.
