# Supplementary material for: Time and frequency analysis of daily-based nexus between global CO2 emissions and electricity generation nexus by novel WLMC approach
Source: Sci Rep. 2024 Feb 14;14:3698. doi: 10.1038/s41598-024-54245-z (PMC10867028; doi:10.1038/s41598-024-54245-z)
Supplement: Supplementary file 1 — Supplementary Information. [file 41598_2024_54245_MOESM1_ESM.docx]

**Time and Frequency Analysis of Daily-Based Nexus between Global CO2 Emissions and Electricity Generation Nexus by Novel WLMC Approach**

**Mustafa Tevfik Kartal^1,2,3,4,*^, Talat Ulussever^5,6^, Ugur Korkut Pata^7,8,9,10^, Serpil Kılıç Depren^11^**

^1^Corresponding Author, European University of Lefke, Department of Banking and Finance, Lefke, Northern Cyprus, Türkiye.

^2^Borsa Istanbul Strategic Planning, Financial Reporting, and Investor Relations Directorate, İstanbul/Türkiye, mustafatevfikkartal@gmail.com, orcid.org/0000-0001-8038-8241.

^3^Lebanese American University Adnan Kassar School of Business, Lebanon.

^4^Azerbaijan State University of Economics (UNEC) Clinic of Economics, Azerbaijan.

^5^Gulf University for Science and Technology, Economics and Finance Department, Kuwait; ulussever@gmail.com, orcid.org/0000-0002-5673-1238.

^6^Research Fellow, Center for Sustainable Energy and Economic Development (SEED), Gulf University for Science and Technology, Kuwait.

^7^Osmaniye Korkut Ata University Department of Economics, 80000 Merkez, Osmaniye/Türkiye, korkutpata@osmaniye.edu.tr, orcid.org/0000-0002-2853-4106.

^8^Lebanese American University Adnan Kassar School of Business, Lebanon.

^9^Azerbaijan State University of Economics (UNEC) Clinic of Economics, Azerbaijan.

^10^European University of Lefke, Department of Banking and Finance, Lefke, Northern Cyprus, Türkiye

^11^Yildiz Technical University Department of Statistics, İstanbul/Türkiye, serkilic@yildiz.edu.tr, orcid.org/0000-0003-4737-7131.

**SI Fig. 1. Trends of the Variable**

**SI Table 1. Descriptive Statistics of the Variables**

| **Variable** | **Mean** | **Median** | **Maximum** | **Minimum** | **Std. Dev.** | **JB Prob.** | **Obs.** |
| --- | --- | --- | --- | --- | --- | --- | --- |
| CO_2_ | 96.51 | 96.14 | 116.12 | 69.00 | 8.31 | 0.0070 | 1186 |
| FOSSIL | 34,428.64 | 34,432.44 | 44,083.46 | 23,506.93 | 4,019.35 | 0.0005 | 1186 |
| RENEW | 17,941.26 | 17,867.05 | 22,969.25 | 12,244.37 | 1,635.75 | 0.0003 | 1186 |
| NUCLEAR | 8,181.46 | 8,160.25 | 9,635.44 | 7,128.93 | 605.14 | 0.0000 | 1186 |
| HYDRO | 9,258.77 | 8,928.92 | 12,432.74 | 6,938.83 | 1,228.09 | 0.0000 | 1186 |
| SOLAR | 2,668.16 | 2,600.93 | 4,423.94 | 1,107.97 | 779.47 | 0.0000 | 1186 |
| WIND | 6,014.32 | 5,918.78 | 10,706.79 | 2,954.64 | 1,389.80 | 0.0000 | 1186 |
| COAL | 23,421.45 | 23,375.63 | 30,229.75 | 16,274.55 | 2,909.06 | 0.0000 | 1186 |
| GAS | 10,566.72 | 10,572.31 | 14,668.11 | 6,899.07 | 1,470.90 | 0.0022 | 1186 |
| OIL | 440.47 | 432.10 | 825.04 | 275.92 | 62.63 | 0.0000 | 1186 |

**Notes**: Std. Dev. denotes Standard Deviation; JB denotes Jarque-Bera Probability; Obs. denotes observations.

**SI Table 2. Correlations between 2020/1-2023/3**

| **Variable** | **CO_2_** | **FOSSIL** | **RENEW** | **NUCLEAR** | **HYDRO** | **SOLAR** | **WIND** | **COAL** | **GAS** | **OIL** | **TELEC** |
| --- | --- | --- | --- | --- | --- | --- | --- | --- | --- | --- | --- |
| CO_2_ | 1.00 |  |  |  |  |  |  |  |  |  |  |
| FOSSIL | 0.80 | 1.00 |  |  |  |  |  |  |  |  |  |
| RENEW | 0.04 | 0.08 | 1.00 |  |  |  |  |  |  |  |  |
| NUCLEAR | 0.67 | 0.46 | -0.36 | 1.00 |  |  |  |  |  |  |  |
| HYDRO | -0.30 | 0.09 | 0.45 | -0.42 | 1.00 |  |  |  |  |  |  |
| SOLAR | -0.22 | -0.04 | 0.71 | -0.62 | 0.39 | 1.00 |  |  |  |  |  |
| WIND | 0.44 | 0.03 | 0.38 | 0.29 | -0.58 | -0.07 | 1.00 |  |  |  |  |
| COAL | 0.81 | 0.96 | 0.10 | 0.52 | -0.03 | -0.04 | 0.17 | 1.00 |  |  |  |
| GAS | 0.56 | 0.82 | 0.01 | 0.22 | 0.31 | -0.01 | -0.25 | 0.62 | 1.00 |  |  |
| OIL | 0.42 | 0.51 | -0.17 | 0.44 | 0.10 | -0.39 | -0.07 | 0.40 | 0.58 | 1.00 |  |
| TELEC | 0.79 | 0.95 | 0.37 | 0.41 | 0.17 | 0.13 | 0.21 | 0.93 | 0.74 | 0.44 | 1.00 |

**Notes**: TELEC denotes total electricity generation from all sources.

**SI Table 3. Correlations for Sub-Periods**

| **Year** | **Variable** | **CO_2_** | **FOSSIL** | **RENEW** | **NUCLEAR** | **HYDRO** | **SOLAR** | **WIND** | **COAL** | **GAS** | **OIL** | **TELEC** |
| --- | --- | --- | --- | --- | --- | --- | --- | --- | --- | --- | --- | --- |
| 2020 | CO_2_ | 1.00 |  |  |  |  |  |  |  |  |  |  |
|  | FOSSIL | 0.82 | 1.00 |  |  |  |  |  |  |  |  |  |
|  | RENEW | -0.21 | -0.05 | 1.00 |  |  |  |  |  |  |  |  |
|  | NUCLEAR | 0.76 | 0.37 | -0.57 | 1.00 |  |  |  |  |  |  |  |
|  | HYDRO | -0.16 | 0.16 | 0.79 | -0.63 | 1.00 |  |  |  |  |  |  |
|  | SOLAR | -0.72 | -0.30 | 0.48 | -0.82 | 0.58 | 1.00 |  |  |  |  |  |
|  | WIND | 0.30 | -0.11 | 0.01 | 0.49 | -0.55 | -0.62 | 1.00 |  |  |  |  |
|  | COAL | 0.82 | 0.95 | -0.14 | 0.45 | -0.01 | -0.36 | 0.02 | 1.00 |  |  |  |
|  | GAS | 0.59 | 0.81 | 0.13 | 0.12 | 0.45 | -0.10 | -0.34 | 0.60 | 1.00 |  |  |
|  | OIL | 0.40 | 0.61 | 0.22 | 0.03 | 0.50 | 0.05 | -0.37 | 0.39 | 0.86 | 1.00 |  |
|  | TELEC | 0.81 | 0.97 | 0.15 | 0.33 | 0.29 | -0.26 | -0.05 | 0.91 | 0.81 | 0.64 | 1.00 |
| 2021 | CO_2_ | 1.00 |  |  |  |  |  |  |  |  |  |  |
|  | FOSSIL | 0.74 | 1.00 |  |  |  |  |  |  |  |  |  |
|  | RENEW | -0.37 | -0.18 | 1.00 |  |  |  |  |  |  |  |  |
|  | NUCLEAR | 0.77 | 0.70 | -0.36 | 1.00 |  |  |  |  |  |  |  |
|  | HYDRO | -0.36 | 0.21 | 0.59 | -0.25 | 1.00 |  |  |  |  |  |  |
|  | SOLAR | -0.68 | -0.34 | 0.57 | -0.73 | 0.65 | 1.00 |  |  |  |  |  |
|  | WIND | 0.30 | -0.21 | 0.15 | 0.22 | -0.67 | -0.53 | 1.00 |  |  |  |  |
|  | COAL | 0.72 | 0.95 | -0.16 | 0.70 | 0.18 | -0.35 | -0.16 | 1.00 |  |  |  |
|  | GAS | 0.63 | 0.89 | -0.18 | 0.57 | 0.22 | -0.28 | -0.25 | 0.71 | 1.00 |  |  |
|  | OIL | 0.43 | 0.70 | 0.06 | 0.34 | 0.29 | -0.14 | -0.15 | 0.58 | 0.72 | 1.00 |  |
|  | TELEC | 0.67 | 0.95 | 0.11 | 0.67 | 0.34 | -0.24 | -0.10 | 0.92 | 0.83 | 0.70 | 1.00 |
| 2022 | CO_2_ | 1.00 |  |  |  |  |  |  |  |  |  |  |
|  | FOSSIL | 0.75 | 1.00 |  |  |  |  |  |  |  |  |  |
|  | RENEW | -0.37 | -0.22 | 1.00 |  |  |  |  |  |  |  |  |
|  | NUCLEAR | 0.83 | 0.50 | -0.46 | 1.00 |  |  |  |  |  |  |  |
|  | HYDRO | -0.28 | 0.12 | 0.78 | -0.49 | 1.00 |  |  |  |  |  |  |
|  | SOLAR | -0.66 | -0.24 | 0.71 | -0.78 | 0.78 | 1.00 |  |  |  |  |  |
|  | WIND | 0.23 | -0.27 | 0.05 | 0.41 | -0.53 | -0.55 | 1.00 |  |  |  |  |
|  | COAL | 0.77 | 0.96 | -0.27 | 0.60 | 0.00 | -0.34 | -0.13 | 1.00 |  |  |  |
|  | GAS | 0.56 | 0.89 | -0.10 | 0.22 | 0.34 | -0.01 | -0.49 | 0.73 | 1.00 |  |  |
|  | OIL | 0.78 | 0.61 | -0.38 | 0.70 | -0.30 | -0.66 | 0.24 | 0.62 | 0.46 | 1.00 |  |
|  | TELEC | 0.71 | 0.95 | 0.08 | 0.47 | 0.32 | -0.10 | -0.17 | 0.91 | 0.84 | 0.55 | 1.00 |
| 2023 | CO_2_ | 1.00 |  |  |  |  |  |  |  |  |  |  |
|  | FOSSIL | 0.92 | 1.00 |  |  |  |  |  |  |  |  |  |
|  | RENEW | 0.36 | 0.22 | 1.00 |  |  |  |  |  |  |  |  |
|  | NUCLEAR | 0.70 | 0.76 | 0.18 | 1.00 |  |  |  |  |  |  |  |
|  | HYDRO | 0.79 | 0.72 | 0.38 | 0.56 | 1.00 |  |  |  |  |  |  |
|  | SOLAR | -0.54 | -0.65 | -0.07 | -0.88 | -0.43 | 1.00 |  |  |  |  |  |
|  | WIND | 0.34 | 0.28 | 0.90 | 0.40 | 0.24 | -0.39 | 1.00 |  |  |  |  |
|  | COAL | 0.78 | 0.87 | 0.52 | 0.77 | 0.62 | -0.64 | 0.61 | 1.00 |  |  |  |
|  | OIL | 0.49 | 0.49 | -0.18 | 0.64 | 0.50 | -0.65 | -0.05 | 0.23 | 1.00 |  |  |
|  | GAS | 0.48 | 0.49 | -0.47 | 0.16 | 0.34 | -0.16 | -0.52 | -0.02 | 0.56 | 1.00 |  |
|  | TELEC | 0.91 | 0.92 | 0.57 | 0.77 | 0.75 | -0.63 | 0.61 | 0.94 | 0.38 | 0.18 | 1.00 |

**Notes**: TELEC denotes total electricity generation from all sources.

**SI Fig. 2. Empirical Process**

**SI Table 4. Stationarities and Linearities of the Variables**

| **Panel** | **Variable** | | **Probability at Level** | | | **Break Date** | | | **Decision** | |
| --- | --- | --- | --- | --- | --- | --- | --- | --- | --- | --- |
| **Panel A:**  **Stationarity** | CO_2_ | | 0.0936 | | | 10/05/2020 | | | I(1) | |
|  | FOSSIL | | 0.0665 | | | 01/24/2022 | | | I(1) | |
|  | RENEW | | 0.0104 | | | 07/26/2022 | | | I(0) | |
|  | NUCLEAR | | 0.0005 | | | 03/07/2022 | | | I(0) | |
|  | HYDRO | | 0.0298 | | | 04/28/2021 | | | I(0) | |
|  | SOLAR | | 0.0495 | | | 02/01/2022 | | | I(0) | |
|  | WIND | | 0.0794 | | | 05/26/2021 | | | I(1) | |
|  | COAL | | 0.0632 | | | 10/07/2020 | | | I(1) | |
|  | GAS | | 0.3193 | | | 09/13/2022 | | | I(0) | |
|  | OIL | | 0.0039 | | | 02/01/2022 | | | I(0) | |
|  |  | **Dimensions** | | | | | | | | **Decision** |
|  |  | **2** | | **3** | **4** | | **5** | **6** | |  |
| **Panel B:**  **Linearity** | CO_2_ | 0.0000 | | 0.0000 | 0.0000 | | 0.0000 | 0.0000 | | NL |
|  | FOSSIL | 0.0000 | | 0.0000 | 0.0000 | | 0.0000 | 0.0000 | | NL |
|  | RENEW | 0.0000 | | 0.0000 | 0.0000 | | 0.0000 | 0.0000 | | NL |
|  | NUCLEAR | 0.0000 | | 0.0000 | 0.0000 | | 0.0000 | 0.0000 | | NL |
|  | HYDRO | 0.0000 | | 0.0000 | 0.0000 | | 0.0000 | 0.0000 | | NL |
|  | SOLAR | 0.0000 | | 0.0000 | 0.0000 | | 0.0000 | 0.0000 | | NL |
|  | WIND | 0.0000 | | 0.0000 | 0.0000 | | 0.0000 | 0.0000 | | NL |
|  | COAL | 0.0000 | | 0.0000 | 0.0000 | | 0.0000 | 0.0000 | | NL |
|  | GAS | 0.0000 | | 0.0000 | 0.0000 | | 0.0000 | 0.0000 | | NL |
|  | OIL | 0.0000 | | 0.0000 | 0.0000 | | 0.0000 | 0.0000 | | NL |

**Notes**: Values shows the p-values. NL denotes the nonlinear

**SI Table 5. PSS & KS Bounds Test for Models**

| **Model** | **K** | | **10%** | | **5%** | | **1%** | | **p-value** | |
| --- | --- | --- | --- | --- | --- | --- | --- | --- | --- | --- |
|  |  |  | **I(0)** | **I(1)** | **I(0)** | **I(1)** | **I(0)** | **I(1)** | **I(0)** | **I(1)** |
| 1 | F | 4.205 | 2.002 | 3.077 | 2.435 | 3.608 | 3.396 | 4.745 | 0.000 | 0.000 |
|  | t | -3.835 | -1.616 | -2.977 | -1.940 | -3.310 | -2.565 | -3.932 | 0.000 | 0.000 |
| 2 | F | 5.725 | 1.741 | 2.862 | 2.035 | 3.233 | 2.663 | 4.002 | 0.000 | 0.000 |
|  | t | -5.912 | -1.616 | -3.735 | -1.940 | -4.072 | -2.566 | -4.696 | 0.000 | 0.000 |
| 3 | F | 3.765 | 2.001 | 3.077 | 2.435 | 3.608 | 3.396 | 4.746 | 0.005 | 0.004 |
|  | T | -3.784 | -1.616 | -2.976 | -1.940 | -3.310 | -2.565 | -3.931 | 0.000 | 0.015 |
| 4 | F | 4.673 | 2.003 | 3.077 | 2.436 | 3.608 | 3.397 | 4.745 | 0.001 | 0.011 |
|  | t | -4.232 | -1.617 | -2.978 | -1.940 | -3.311 | -2.565 | -3.933 | 0.000 | 0.004 |
| 5 | F | 11.108 | 2.002 | 3.077 | 2.436 | 3.608 | 3.397 | 4.745 | 0.000 | 0.000 |
|  | t | -6.631 | -1.616 | -2.978 | -1.940 | -3.311 | -2.565 | -3.932 | 0.000 | 0.000 |

**Notes**: Where I(0) and I(1) denote the lower and upper band critical values at 10%, 5% and 1% significance level of Pesaran, Shin, and Smith bounds test; P-value is Kripfganz & Schneider critical values and approximate p-values.

**SI Table 6. Baseline Regression Estimations by the DOLS Approach**

| **Variable** | **Models** | | | | |
| --- | --- | --- | --- | --- | --- |
|  | **1** | **2** | **3** | **4** | **5** |
| FOSSIL |  | 0.0011 [0.0000] |  |  |  |
| RENEW |  | 0.0006 [0.0000] |  |  |  |
| NUCLEAR | 0.0026 [0.0000] | 0.0059 [0.0000] |  |  | 0.0057 [0.0000] |
| HYDRO | 0.0012 [0.0002] |  |  | 0.0056 [0.0000] | 0.0006 [0.0041] |
| SOLAR | -0.0021 [0.0001] |  |  | -0.0037 [0.0000] |  |
| WIND | 0.0042 [0.0000] |  |  | 0.0091 [0.0000] |  |
| COAL | 0.0015 [0.0000] |  | 0.0035 [0.0000] |  | 0.0024 [0.0000] |
| GAS | 0.0017 [0.0000] |  | -0.0007 [0.2602] |  |  |
| OIL | -0.0187 [0.0031] |  | 0.0474 [0.0000] |  |  |
| R^2^ | 93.68 | 78.09 | 63.75 | 55.78 | 81.93 |
| Adj. R^2^ | 91.65 | 77.83 | 58.80 | 49.74 | 79.47 |

**Notes**: [ ] denotes probability values.

**SI Table 7. Time Schedule for the WLMC Figures**

| **Number of Observation** | **Exact Date** |
| --- | --- |
| First | 01.01.2020 |
| 200th | 18.07.2020 |
| 400th | 03.02.2021 |
| 600th | 22.08.2021 |
| 800th | 10.03.2022 |
| 1000th | 26.09.2022 |
| Last | 31.03.2023 |

**SI Table 8. Frequencies for the WLMC Figures**

| **Frequency Interval** | **Frequency Term** |
| --- | --- |
| 2-16 | Short-Term |
| 16-64 | Medium-Term |
| 64-128 | Long-Term |
| 128-256 | Very Long-Term |
